# HOUSING ATTRIBUTE PREFERENCES WHEN CONSIDERING RELOCATION IN OLDER AGE

**DOI:** 10.1093/geroni/igad104.0736

**Published:** 2023-12-21

**Authors:** Maya Kylén, Björn Slaug, Susanne Iwarsson, David Dahlgren, Jonas Bjork, Magnus Zingmark

**Affiliations:** Lund University, Lund, Skane Lan, Sweden; Lund University, Lund, Skane Lan, Sweden; Lund University, Lund, Skane Lan, Sweden; Lund University, Lund, Skane Lan, Sweden; Skane University Hospital, Lund, Skane Lan, Sweden; Lund University, Lund, Skane Lan, Sweden

## Abstract

For the last decades, interdisciplinary researchers have studied housing preferences of various groups in the population. To contribute to knowledge on the needs and preferences of the aging population we have established a cohort (N=1,509) of adults, 55 years or older, who are considering relocation. The baseline survey included questions on the current housing situation and preferences regarding future housing alternatives. The aim was to investigate preferred housing attributes when considering relocation and whether such attributes differed depending on the type of housing participants wanted to move to. Preliminary results show that having a balcony/patio was rated as most important (84%), with significant differences between men and women. Leisure activities and/or social gatherings initiated by the landlord was the least important (1.5%). Differences in preferred attributes were found between those wanting to move to privately-owned housing (e.g., villa), where having a garden (81%) or a view (57%) were the most important attributes, and those wanting to move to an apartment, where having a balcony (86%) or lift (72.6%) were deemed most important. We expect that the longitudinal follow up of the cohort will contribute with new knowledge on housing preferences among people in later life. Such knowledge is important to inform and inspire planners and policymakers to develop more age-friendly communities, including housing options that better meet the needs of this segment of the population.

